# Performance of Fly Ash-Based Inorganic Polymer Mortar with Petroleum Sludge Ash

**DOI:** 10.3390/polym13234143

**Published:** 2021-11-27

**Authors:** Mubarak Usman Kankia, Lavania Baloo, Nasiru Danlami, Bashar S. Mohammed, Sani Haruna, Mahmud Abubakar, Ahmad Hussaini Jagaba, Khalid Sayed, Isyaka Abdulkadir, Ibrahim Umar Salihi

**Affiliations:** 1 Civil and Environmental Engineering Department, Universiti Teknologi PETRONAS, Seri Iskandar 32610, Perak, Malaysia; mubarak_18001828@utp.edu.my (M.U.K.); bashar.mohammed@utp.edu.my (B.S.M.); sani_17000823@utp.edu.my (S.H.); ahmad_19001511@utp.edu.my (A.H.J.); khalid_19000239@utp.edu.my (K.S.); isyaka_18000638@utp.edu.my (I.A.); 2 Civil Engineering Department, Bayero University, Kano PMB 3011, Nigeria; nassday@live.com (N.D.); iusalihi@gmail.com (I.U.S.); 3 Civil Engineering Department, Federal University of Technology, Minna PMB 65, Nigeria; mahmud1879@futminna.edu.ng

**Keywords:** petroleum sludge ash, fly ash, geopolymer mortar, mechanical properties, microstructural properties, response surface methodology

## Abstract

Petroleum sludge is a waste product resulting from petroleum industries and it is a major source of environmental pollution. Therefore, developing strategies aimed at reducing its environmental impact and enhance cleaner production are crucial for environmental mortar. Response surface methodology (RSM) was used in designing the experimental work. The variables considered were the amount of petroleum sludge ash (PSA) in weight percent and the ratio of sodium silicate to sodium hydroxide, while the concentration of sodium hydroxide was kept constant in the production of geopolymer mortar cured at a temperature of 60 °C for 20 h. The effects of PSA on density, compressive strength, flexural strength, water absorption, drying shrinkage, morphology, and pore size distribution were investigated. The addition of PSA in the mortar enhanced the mechanical properties significantly at an early age and 28 days of curing. Thus, PSA could be used as a precursor material in the production of geopolymer mortar for green construction sustainability. This study aimed to investigate the influence of PSA in geopolymer mortar.

## 1. Introduction 

Due to the population growth and modern construction standards demand more infrastructures; thus, concrete and mortar utilizations are significantly increasing to produce most of the elements. Portland cement (PC) is the main source material used as a binder for the clean production of both mortars, reinforced, and plain concrete, which has a significant consumption [1]. PC is manufactured through grinding and calcinating processes using raw materials such as clay, limestone, etc. and the temperature of calcination can reach up to 1450 °C. PC production is associated with relatively high resources and energy consumption as well as environmental problems [2]. PC production keeps increasing yearly by 9% globally and this increase results in severe problems to the environment because of the high volume of CO_2_ discharged into the surrounding during its manufacture [3]. Statistical trends estimated an increase in cement production globally from ~4.3 billion metric tons in 2015 to ~6.1 billion metric tons in 2050 [4]. This rate is even higher in developing countries such as China with a production of about half of the global cement in 2019 [5]. However, one ton of PC emits approximately one ton of carbon dioxide (CO_2_) into the environment [6]. Precisely, about 1.5 billion tons of greenhouse gases are released annually during the manufacture of OPC, or approximately 6% of the total global greenhouse gas emission [7,8]. One of the effects of the greenhouse is the prevention of solar radiation reflected into space; thus, maintaining consistent temperature on the surface of the earth from 15 °C to 18 °C [9]. Also, the emission affects the natural phenomenon which accounts for approximately 65% of global warming [10]. Sustainable development of new low carbon binding materials to replace PC which can limit the emissions of CO_2_ and decrease global warming has attracted great interest in seeking solutions as the world faces continuous environmental degradation [1,9].

Geopolymer is an innovation [11] and it is a form of cement-less binding material as well as an appropriate green construction material utilized as a sustainable low carbon alternative to PC depending upon its application; providing many opportunities to use either agricultural or industrial wastes with promising properties in the sector of construction. Materials that may be manufactured from the amorphous mineral powder rich in aluminates and silicates react relatively slow with water but react through condensation and hydrolysis in an alkaline activation solution to produce inorganic polymers that can resist loads [12]. Geopolymer products are cured at an elevated temperature ranging from 60 °C to 100 °C with a duration of 20 to 36 h to enhance the geopolymerization reaction [13,14]. The geopolymerization reaction process can be grouped into several steps: (i) solid aluminosilicate materials dissolution in an extremely concentrated alkaline solution; (ii) silica-alumina oligomers formation; (iii) the oligomeric species poly-condensate to produce an inorganic polymeric material; and (iv) undissolved solid particles bond together in the final structure of geopolymer [15]. Geopolymers have many applications due to their good performance characteristics; geopolymers can be used to immobilize toxic waste materials, coat radioactive materials, geopolymers also resist fire and high temperature, etc. [16]. Geopolymer technology also offers benefits in recycling waste materials as significant binders. Many studies [17,18,19,20] have been performed to examine the feasibility of industrial by-products as the precursor materials of geopolymer. Until now, much industrial waste has been proven to be suitable for geopolymer synthesis. In recent years, geopolymer production involves the utilization of precursor materials rich in amorphous SiO_2_ and Al_2_O_3_ including agricultural waste ashes such as palm oil fuel ash, rice husk ash, corn cob ash, straw ash, sugarcane bagasse ash, wood ash, forest biomass bottom ash; industrial waste ashes such as coal bottom ash, fly ash, silica fume, industrials slags, artificial pozzolans (ceramic residues, calcined clays, sedimentary rocks containing burned bauxites and clay minerals); the ash produced from municipal solid waste like sludge ash, glass powder, and municipal solid waste incinerator bottom fly ash were used as source materials to manufacture geopolymers [15,21].

Alkaline activators like potassium hydroxide (KOH), sodium hydroxide (NaOH), potassium silicate (K_2_SiO_3_), and sodium silicate (Na_2_SiO_3_) are used for the activation of these aluminosilicates (precursor) materials. In the concentrated alkaline solution, precursors are dissolved rapidly and release alumina [AlO_4_]^−^ and silica [SiO_4_]- tetrahedral units. These units are alternatively connected forming a polymeric bond that develops its binding features based on the reaction shown in Equation (1) [22].

(Si_2_O_5_ Al_2_O_2_)_n_ + H_2_O + OH → Si(OH)_4_ + Al(OH)^4−^ (1)

Petroleum sludge is a thick and viscous combination of solid residue, waste oil products, hydrocarbons, and water, that is generated during the crude oil refining processes, storage, as well as vessel cleaning [23,24,25], and it is produced more than 6 million tons annually in China [26,27]. The main areas of producing petroleum sludge are crude oil storage vessels and the separation of water from oil [23]. The petroleum types and sources are the main factors that influence the petroleum sludge complexity [28]. The mixture of petroleum sludge is mainly generated due to sedimentation and accumulation of crude oil in the storage tanks [29]. Petroleum sludge consists of petroleum hydrocarbons like aromatic, asphaltene, aliphatic, and components of nitrogen sulfur-oxygen [25,30]. It is characterized by alkanes 40–52%, aromatics 28–31%, resins 7–22.5%, and asphaltenes 8– 10% [31]. It consists of 30–40% water, 10–20% solid particles, and 30–80% oil by mass [32]. It is generally considered hazardous waste due to the presence of harmful compounds, heavy metals, solid particles, as well as persistent and recalcitrant sediments [30,31,32,33]. Thus, petroleum sludge treatment is necessary to prevent soil pollution and keep its functionality, as well as avoid water contamination in the environment [34]. Several biological, chemical, and physical methods are used to recycle biomass energy [35,36]. Among these techniques, the incineration method [36], stabilization, and solidification have attracted more attention than the others because of their remarkable advantages of decreasing the hazardousness of petroleum sludge.

Pinheiro and Holanda [37] used petroleum sludge in the production of red ceramic products. They found up to 30 wt % of petroleum sludge can be used in red ceramic manufacturing; they reported enhancement of linear shrinkage, apparent density, and water absorption for all firing temperatures; decrease in mechanical strength; proper for clay bricks and roofing tiles. Pinheiro and Holanda [38] partially replaced kaolin material with petroleum sludge up to 5 wt % in the production of porcelain stoneware tile. It was discovered that the replacement of kaolin with petroleum sludge, in the range up to 2.5 wt %, permits the manufacture of porcelain stoneware tiles (group BIa, ISO 13,006 standard); safe leaching limits; the results showed that the addition of more petroleum sludge into tile formulations resulted in a decrease of linear shrinkage, apparent density, and flexural strength, and an increase of water absorption of the produced tile materials. Xiao et al. [39] utilized petroleum sludge as a material for roadbed with fly ash, ordinary Portland cement, and silica fume as binders and phosphogypsum as a stabilizer. The effectiveness of the stabilization and solidification process was assessed primarily through an unconfined compressive strength test and a toxicity leaching test. The results showed that the addition of 20 wt % of binders in combination with phosphogypsum to the petroleum sludge not only increases the 28-day compressive strength of the solidified samples and remarkably decreases the release of heavy metals but also refines the pore structure and compacts the microstructure. Similarly, Souza et al. [40] incorporated petroleum sludge in the production of clay-based ceramics (hollow bricks and roofing tiles) up to 30 wt % of waste as a replacement for natural clay, and the result showed that the heavy metals are within safe leaching limits. Furthermore, Sengupta et al. [41] utilized the petroleum sludge in preparing environmentally acceptable masonry bricks in a commercial brick plant. The addition of the sludge reduced the required fuel and water in the production process. The fired bricks met all the requirements of the Indian Standard.

The blend of geopolymer mortar using PSA and fly ash (FA) certainly is worth exploring concerning the feasibility of recycling wastes from a crude oil refinery and a coal power plant to reduce cement usage. Using RSM, the effect of PSA content and the ratio of sodium silicate (NS) to sodium hydroxide (NH, NS: NH) were also studied through the mechanical and microstructural properties of geopolymer mortar (density, unconfined compressive strength (UCS), flexural strength, drying shrinkage of geopolymer mortar, morphology, and pore size distribution respectively). This study encompasses efforts of recycling waste products as the precursor materials for the synthesis of geopolymer mortar which can provide a significant guide to both the industries and academia.

## 2. Material and Methods

### 2.1. Materials

**Figure 1:**
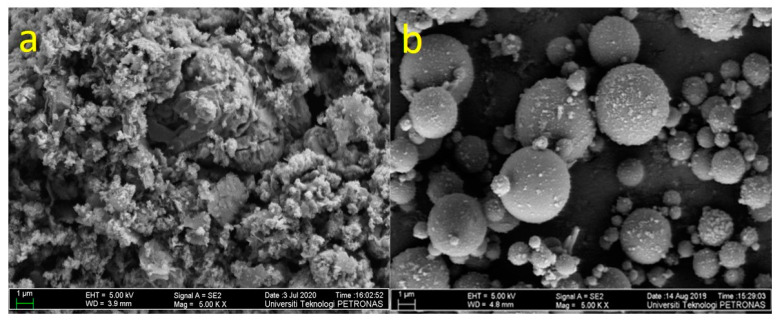
FESEM image of (**a**) PSA (**b**) FA.

The petroleum sludge was collected from a refinery in Malaysia. It appeared to be black, viscous, and semi-solid with an unpleasant smell. The petroleum sludge is classified as scheduled waste (SW314) by the Department of Environment, Malaysia (2010 guidelines). An air suction filter was used to dewater the petroleum sludge and then put it into an oven at 105 °C to dissipate the remaining water in the petroleum sludge for 6 h. Approximately 500 g of the dried petroleum sludge, was mixed with additives 1 mole of sodium bicarbonate (NaHCO_3_) and 1 mole of Calcium hydroxide (Ca(OH)_2_) obtained from Avantis laboratory supply, Perak, Malaysia. The additives assisted in degrading the polycyclic aromatic hydrocarbons [42]. The petroleum sludge was burnt in the laboratory muffle furnace for 2 h at a temperature of 600 °C (most metals volatilize at 650 °C) [43]. PSA was obtained after the burning and had bigger particles and it was milled in a laboratory milling machine and sieved through 45 µm [44]. The chemical composition of the PSA was determined using X-Ray Fluorescence (XRF) and presented in Table 1. The sum of Al_2_O_3_, SiO_2_, and Fe_2_O_3_ for the PSA is 70.80% along with loss on ignition (LOI) of 0.09% and calcium oxide of 9.26%; thus, conforms to the ASTM C618–15 specification [45]. The morphology of PSA is determined using the Field Emission Scanning Electron Microscopy (FESEM) and shown in Figure 1a indicating that the PSA consists of a pack of angular grains. PSA was used as a secondary binder consists mainly of aluminosilicate calcium materials.

**Figure 2:**
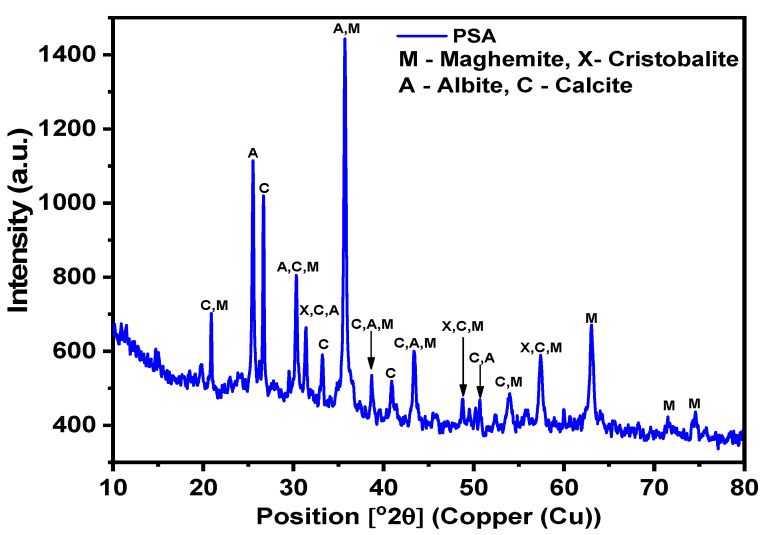
XRD graph of PSA.

In this study, FA of high calcium content was taken from a Manjung coal power plant, Perak, Malaysia. The FA was used as the main source of aluminosilicate material. The pozzolanic oxides of the FA were determined through XRF and presented in Table 1. The total percentage of Al_2_O_3_, Fe_2_O_3_, and SiO_2_ is 74% for FA; thus, it satisfies the requirement of the ASTM 618-10 standard method. The morphology of the FA was determined using FESEM and presented by the micrograph in Figure 1b. The particles of the FA appeared spherical, allowing them to blend in the mixtures. It is worth stating that the content of calcium oxide is greater than 10%. In the present study, FA was replaced with PSA. The NH (purity 99%), and the liquid NS containing Na_2_O 14.37 wt %, SiO_2_ 29.54 wt %, H_2_O 56.09 wt % was obtained from a local supplier. For the manufacture of the geopolymer mortar, locally available natural-siliceous sand having a fineness modulus of 2.52 and agrees to ASTM C33/C33M—13 [46]. The XRD patterns of the PSA are shown in Figure 2. The pattern showed the presence of albite with reference code: ICDD#01–071-3816 and chemical formula: Na(AlSi_3_O_8_); maghemite has reference code: ICDD#00–025-1402 and chemical formula: Fe_2_O_3_ are the major diffraction peaks. Also, smaller diffraction peaks such as calcite and cristobalite were observed.

### 2.2. Methods

#### 2.2.1. Statistical Model Development of Geopolymer Mortar Using Response Surface Methodology

This section explained the series of steps for mixture optimization. The experimental design along with model development, optimization, and validation was done using response surface methodology (RSM). The central composite design method was used for the design of experiments based on two variables (the amount of PSA in the geopolymer mortar, and the ratio of NS to NH). The experimental factors were varied in 3 levels (lower, middle, and upper level respectively). The content of PSA (0%, 10%, and 20% by weight) and the ratios of NS to NH (1.5, 2.0, and 2.5), are depicted in Table 2. Two responses were duly considered which include flexural strength, and water absorption respectively. According to the experimental design from the RSM software, 13 mixtures with five duplications were developed. The mix combinations (independent variables) for the geopolymer mortar are illustrated in Table 3. The P20R2 (mixture number 1) stands for 20 wt % of FA is replaced with PSA, R (NS: NH) = 2.

#### 2.2.2. Mixing, Casting, and Curing of Geopolymer Mortar

The mixed proportions of geopolymer mortar are shown in Table 4. The alkaline solution to binder ratio of 0.5 and sodium hydroxide molarity of 12 were used to prepare the geopolymer mortar specimens. Also, the binder to the sand ratio of 1:2 was adopted [47]. A day before the synthesis of the mortar, NH pellets were dissolved in a calculated quantity of water to produce sodium hydroxide solution and cooled up to ambient temperature following the RSM mix formulations presented in Table 3. Before the experiment, the required amount of NS and NH solutions were homogeneously mixed in a beaker to form the activation solution. The quantities of FA, fine aggregates, and PSA were measured and then mixed thoroughly in a small Hobart mixer for approximately 2 min. Also, the alkaline solution was gently added into the dry mix and then mixed for more than 3 min until it became consistent and homogeneous.

The molds were thoroughly cleaned and tightened, as well as appropriately lubricated before casting. Furthermore, the mixtures were cast into 50 mm cubic molds for the test of compressive strength and water absorption. Shrinkage prisms with 25 mm square cross-section and about 285 mm in length were cast to conduct the shrinkage test. Similarly, flexural beams of 500 × 100 × 25 mm^3^ were cast. The molds were carefully filled and compacted with the aid of the vibrating table for approximately 30 s and the top surface areas were screed off to eliminate excess geopolymer mortar mix. For each mix formulation, three samples were prepared. After 24 h, the hardened mortar samples were removed from the molds. The geopolymer mortar samples were properly placed in an oven and then cured at a temperature of 60 °C for 20 h [48]. The specimens were kept in the laboratory with a relative humidity of about 60% and a temperature of 20 ± 2 °C until the testing days (3, 7, and 28 days).

#### 2.2.3. Tests on the Geopolymer Mortar

The tests conducted on the geopolymer mortar consist of density test, compressive strength, flexural strength, water absorption as well as drying shrinkage test, and test microstructural properties.

##### Hardened Density

The hardened density of geopolymer mortar was determined by weighing the cube samples on the testing day before the compression test. Three cubes for each mix proportion were measured at 3, 7, and 28 days to obtain the density; the weight of the cube was divided by its volume and the average was calculated and reported.

##### Compressive Strength

In this study, a digital compressive testing machine of 3000 kN capacity was used to determine the strength of the geopolymer mortar specimens (cubes, 50 × 50 × 50 mm^3^) according to the standard test method (ASTM C109/C109 M standard) [49] at the age of 3, 7, and 28 days. For every test, the cube was subjected to a force at a loading rate of 0.9 kN/s for the geopolymer mortar sample until it failed. On each day of testing, triple cubes were used to determine the average compressive strength. The strength is obtained by dividing the maximum load with a cross-sectional area.

##### Flexural Strength

Flexural strength, also known as the bending strength or rupture modulus, or transverse rupture strength, is a property of the material that is defined as the stress in a material just before giving way in a test of flexure. It is a degree of resistance of a material to rupture or bending. In this study, the flexural strength test was carried out according to ASTM C293/C293M [50] employing a simple beam with a center point load. The test was done with a universal testing machine (200 kN capacity). Three rectangular specimens with sizes of 500 × 100 × 25 mm^3^ were manufactured for each mixture of geopolymer mortar and used for testing the mortar specimens. The loading rate was applied at 0.1 mm/min for each mortar sample tested. The test was conducted for only 28 days. The average results of the three tested samples were recorded. The force along with the resulting midspan deflection was generated from a computer data device. The maximum load was used to compute the flexural strength using Equation (2).

(2) $$Fs=\frac{3PL}{2bd^{2}}$$

where *Fs* = flexural strength, *P* = maximum load applied (kN), *L* = effective length (mm), *b* = width of the sample (mm), *d* = sample depth in mm.

##### Drying Shrinkage

The decrease in length of the geopolymer mortar samples instigated by water loss during the process of drying is known as drying shrinkage. In this study, the drying shrinkage test was carried out according to the method described in ASTM C157/157M-17 [51]. A digital Vernier caliper was employed to determine the length of the de-molded geopolymer mortar samples. For each mix proportion, three specimens (285 × 25 × 25 mm^3^) were cast. The decrease in length was obtained at 3, 7, 28, 56, and 90 days respectively. The drying shrinkage was computed using Equation (3). The average drying shrinkage of the three specimens was recorded.

(3) $$\mathrm{Drying}\;\mathrm{Shrinkage}\;=\frac{L_{0}-{\;L}_{t}}{L_{0}}\times 100$$

where L_o_ = initial length, L_t_ = measured length at each testing day.

##### Water Absorption

A water absorption test is employed to examine the durability characteristic of geopolymer mortar. Geopolymer mortar can absorb fluid from the surroundings and fill the void spaces in the mortar matrix, whereas porosity refers to the number of pore spaces in the mortar matrix. The test has been performed on hardened mortar according to the specifications of ASTM C642-13 [52]. 50 × 50 × 50 mm^3^ cubes were used for the water absorption test of geopolymer mortar. After curing time has elapsed (28 days), the mortar samples were oven-dried at a temperature of 105 °C for 24-h and the dried weight was recorded. The specimens were dried further in the oven at 105 °C for one day more and cooled as well as weighed again until a constant mass was obtained (constant weight was achieved when differences between two successive masses are less than 0.5 percent). The constant weight was noted as W_1_. The mortar samples were submerged in water for 24 h at room temperature. After the time has elapsed, the mortar specimens were removed from the water. The surfaces of the cubes were dried with the help of a towel. They were weighed again and recorded as W_2_. Water absorption was calculated using Equation (4).

(4) $$Water\;absorption=\frac{W_{2}-{\;W}_{1}}{W_{1}}\;\times \;100$$

##### Microstructural Properties of Geopolymer Mortar

A field emission scanning electron microscope (FESEM) was used to examine the crack surface features of the developed geopolymer mortar. FESEM is said to be an ultra-high-resolution microscope that utilizes a focused electron beam for scanning specimens by producing an image of the samples. The test was conducted on the fly ash-based geopolymer mortar with the inclusion of PSA to study the variations in surface morphology of the mortars. The test was conducted according to ASTM C1723—10. Before the test, sample preparation was done by coating it with 200 A ° Gold-Palladium with a sputter coater model. Carl Zeiss ultra-high resolution SUPRA 66VP by Carl Zeiss AG, Germany was employed to observe the micrographs of the geopolymer mortar.

Mercury intrusion porosimetry (MIP) test is an excellent technique employed to assess the pore distribution, porosity, and volume of pores of several powders and solid materials. The test of MIP has been utilized to investigate the pore distributions along with pore sizes for geopolymer mortar. The test was conducted according to the standard procedures in ASTM D4284. Thermo-fisher scientific porosimeter attached to a computer data acquisition system fitted with software was used to retrieve the measurements of pore from the porosimeter. Three mortar specimens were chosen according to PSA content. The samples were prepared by crushing the mortar cubes (about 10 mm). Before the test, the specimens were preconditioned to take away any contamination from the pores and pore walls. This was conducted by outgassing the mortar samples for approximately 8 h at a temperature of 105 ºC in a less pressure vacuum of about 1.3 Pa.

##### Polycyclic Aromatic Hydrocarbons of Geopolymer Mortar

Geopolymer mortar samples containing 10% and 20% of PSA in the mixture were used for this study. Soxhlet extraction for 24 h in a mixture of hexane-dichloromethane (1:1) was conducted according to the 3540C method of U.S. EPA [53]. Polycyclic aromatic hydrocarbons (PAHs) were analyzed by gas chromatography-mass spectrometry (GC-MS) in the scan mode in an Agilent system (6890 gas chromatography coupled with a 5973-mass spectrometer), with an HP5-MS column (30 m × 0.25 mm i.d. × 0.25 mm) using USEPA method 8270C as reference. The identification with quantification was conducted for the established 16 priority PAHs by the USEPA.

## 3. Results and Discussion

### 3.1. Density of Geopolymer Mortar

**Figure 3:**
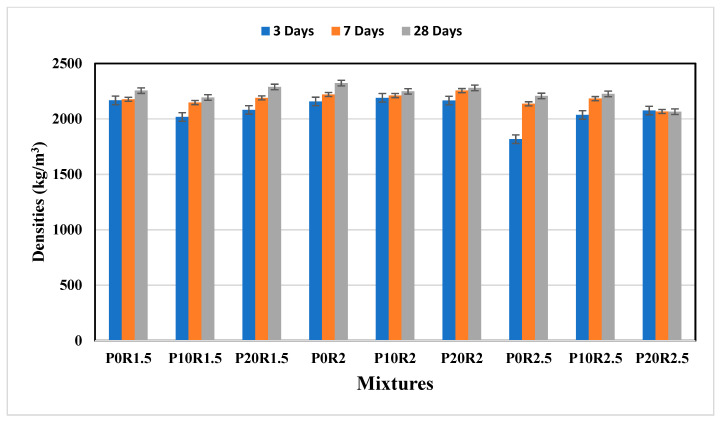
Densities of geopolymer mortar samples.

Figure 3 illustrated the densities of the geopolymer mortars at the different compositions of PSA. The present study unveiled that the developed densities of the geopolymer mortars increased slightly with age, which is attributed to the moderate increase in the reaction of geopolymerisation at 14 and 28 days, thus resulted in pore refinement and compact matrices. The hardened densities range from 1817.33 kg/m^3^ to 2322.95 kg/m^3^, which are higher than the densities reported by Yahya et al. [54] and are below the limit (2400 kg/m^3^). At 28 days, the mixture made with 0 wt % of PSA and 2 as the ratio of NS to NH (P0R2) has a higher density of about 2322.95 kg/m^3^ than P10R2 and P20R2 by 3.21% and 18.61%. This may be attributed to the angular grains of PSA in the geopolymer mortar mixtures resulted in the less compact network structures. As the ratio of NS to NH increases from 1.5 to 2, the density increased accordingly. However, a further increase in the ratio resulted in a decrease in density.

### 3.2. Compressive Strength Development

**Figure 4:**
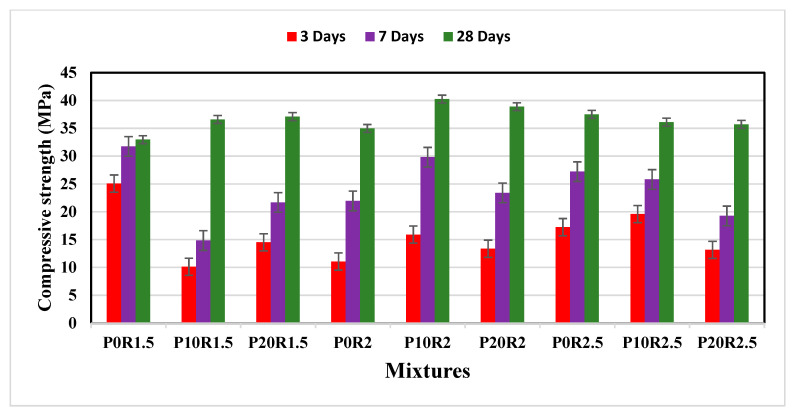
Compressive strength growth of geopolymer mortar.

The growth of compressive strengths of various geopolymer mortar mixture combinations was evaluated at 3, 7, and 28 days. Fly ash was substituted with PSA at 0%, 10%, and 20%. At 3 days, there is a significant strength gain of the geopolymer mortars, which is attributed to the existence of alkali in the matrix that promotes the process of geopolymerization at an early age. Furthermore, the compressive strength increased with the age of curing of 3 to 28 days. This may be attributed to the reaction of PSA particles in the geopolymer mortar matrix, and the continuity of the geopolymerization reaction process at later ages, which resulted in pores refinement, as well as the multi-condensation of the aluminosilicate in the mixture, resulting in producing three-dimensional structures and forming calcium aluminosilicate hydrate gel (C–A–S–H). The present calcium content in the source materials helps developing compact and dense mortar structures. Compared with the reference mortar (P0R1.5), Figure 4 shows that the compressive strength increases as the percentage of PSA increase for the geopolymer mortar mixtures with 1.5 as the ratio of NS to NH (R). This may be attributed to the dissolution of alumina and silica which improved the geopolymerization process. When R is 2, the 28 days compressive strengths of P10R2 and P20R2 were found to be higher than that of the control geopolymer mortar (P0R2) by 13.11% and 10.05%. The 28-day compressive strength of the reference geopolymer mortar P0R2.5 is higher than that of the corresponding mortars P10R2.5 and P20R2.5 by 3.73% and 4.77%. This decrease could be attributed to the excess of the amount of PSA and alkaline solution (OH^−^), which prevents the geopolymerization reaction process and weakens the mortar bond. The decrease in compressive strength showed lesser geopolymerization. Several researchers [55,56,57,58] reported that the proportion of fly ash with biomass ash to acquire a geopolymer with moderately high compressive strength (between 20 MPa to 50 MPa) was found to be within 65% and 35%. The 28 days compressive strengths of P0R2 and P0R2.5 were found to be higher than that of P0R1.5 by 6.10% and 13.77%. Similarly, comparing with P10R1.5, the mixture P10R2 has a compressive strength of 40.25 MPa, which is higher by 10% while the compressive strength of P10R2.5 decreased by 1.33%. Furthermore, the compressive strength of P20R2 increased by 4.80% and the strength of P20R2.5 is lower than that of P20R1.5 by 3.75%.

### 3.3. Influence of PSA Content and Na_2_SiO_3_: NaOH Ratio on Flexural Strength

**Figure 5:**
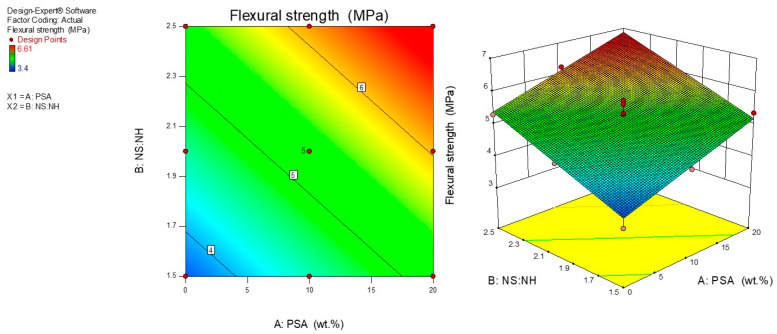
2-D and 3-D plots for flexural strength of geopolymer mortar.

Figure 5 presents the flexural strength of fly ash and PSA geopolymer mortars at 28 days. The geopolymer mortars were cured at 60 °C for 24 h followed by keeping the mortar samples at room temperatures until the testing day. The flexural strength of the mortar samples was improved when the PSA was added to the mixtures. Furthermore, the ratios of sodium silicate to sodium hydroxide have considerable effects on the development of the geopolymer flexural strength. It was observed that with the increase in the ratio from 1.5 to 2.5, the flexural was improved.

The 2-D surface graph in Figure 5 shows the variation of the flexural strength response model for the input variables considered. It is seen that the whole contour lines were linear in shape displaying the interactions between the input variables. The model was found to have a good correlation between the independent variables. It can be observed that the PSA contributed significantly towards the improvement of the flexural strength of the geopolymer mortar. It is interesting to note that the flexural strength of the geopolymer mortar increases with the increase in the ratio of sodium silicate to sodium hydroxide (NS: NH) at a given PSA content. The minimum flexural strength is obtained in the bluish portion while the optimum is at the yellowish area of the contour graph. The 3-D graph is shown in Figure 5 displayed that all the input factors have a significant influence on the flexural strength of the geopolymer mortar linearly. The flexural strength of the mortar was enhanced when all the input variables were increased. However, the flexural strength was reduced when the content of PSA and the ratio of NS to NH ratios were decreased. The mortar combination of P20R2.5 and P0R1.5 yielded the maximum flexural strength of 6.61 MPa and the minimum flexural strength of 3.4 MPa.

### 3.4. Influence of PSA Content and Na_2_SiO_3_: NaOH Ratio on Water Absorption

**Figure 6:**
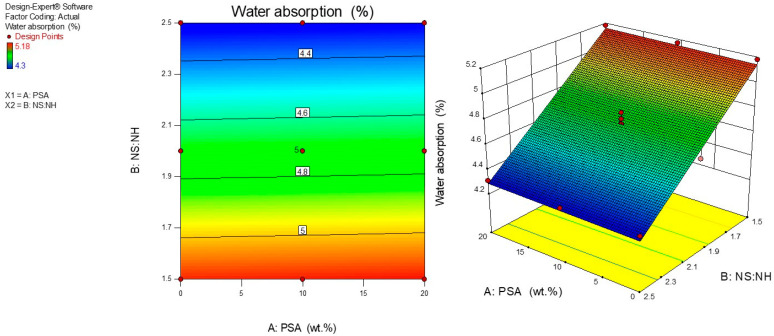
Water absorption of geopolymer mortar at 28 days.

The use of a water absorption test is to evaluate the performance of the geopolymer mortar made with and without PSA and the influence of the NS to NH ratio. Figure 6 displays the 2-D contour plots and 3-D diagram for the water absorption model of fly ash with PSA geopolymer mortar. It was observed that the contour lines were linear in shape showing an ideal interaction between the ratio of NS to NH and the amount of PSA. The established model was found to display an ideal synergy between the input variables. The yellowish to bluish portions in Figure 6 showed an outstanding combination that produces minimum water absorption values. It is interesting to note that the water absorption decreased proportionately with the increase in the ratio of NS to NH for a given content of the PSA. The bluish areas on the contour plots show the region of the least water absorption of the modified geopolymer mortar whereas the reddish part designates a portion of relatively higher water absorption. The 3-D response surface graph explaining the effects of the input variables on water absorption of the mortar is presented in Figure 6. It is seen that the 3-D surface plot revealed that two variables affected the water absorption significantly. As shown in Figure 6, the water absorption values were obtained between 4.3% and 5.17% which are similar to the values reported by Atabey et al. [59]. The water absorption decreased linearly with an increase in both the ratio of sodium silicate to sodium hydroxide (NS: NH) and PSA content. This could be associated with the viscous solution, enhancement of homogeneous structure of the mortar matrices, and improvement of the pores in the geopolymer mortars resulting in a rapid geopolymerization reaction process that increased the densification of the mortar matrix preventing water infiltration. Also, heat curing at 60 °C for 20 h decreased the volume of voids in the geopolymer matrices which is in line with the findings of Noushini and Castel [60].

### 3.5. Drying Shrinkage of Geopolymer Mortar

**Figure 7:**
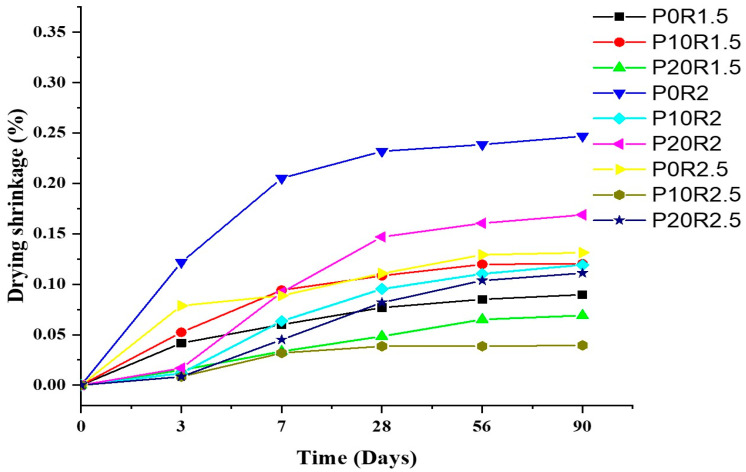
Drying shrinkage of geopolymer mortar.

The arithmetic mean values of drying shrinkage of the geopolymer mortars were recorded at the 3, 7, 28, 56, and 90 days as depicted in Figure 7. The geopolymer mortar drying shrinkage occurs because of the moisture removal from the exterior of its gel pores, thus producing volume changes. Unlike mortar made with OPC, the mixing water appears not to directly be incorporated in the production of geopolymer gel; moreover, a small amount of the mixing water lives as interstitial water in the gel of geopolymer [61]. Conversely, a considerable amount of water is released, and a little volume of mixing water is chemically bound, which may dissipate under lower conditions of relative humidity at room temperature. Hence, loss of redundant water because of evaporation can increase drying shrinkage in geopolymer mortars if compared to OPC mortars [62]. While the process of drying, the water in the geopolymer mortar mixtures directs to stream from the big pore to a less pore due to the gradient pressure. When the meniscus penetrates the big pore, the dimension of the meniscus within the holes with the radius cannot be further reduced, hence, the big pore begins to drain. Also, in the smaller pores, the evaporation results in a reduction in the size of the meniscus [63].

Mortar drying shrinkage is essentially caused by the binder form and may start debonding in the paste/aggregate inter-faces, hence, result in the growth of intergranular cracks [64]. Therefore, it is constantly better to have a mortar mixture with decreased drying shrinkage. As depicted in Figure 7, the drying shrinkage rates were defined by high initial growth, approximately reaching about 50% of 90 days at first 28 days for most of the geopolymer mortar mixture combinations. The shrinkage became almost stabilized after 28 days and stated that 89% of drying shrinkage could be reached within 28 days for geopolymer samples. Consequently, the 28-day value of shrinkage is accepted for studying the impact of various parameters [65]. Reference to the control sample of geopolymer mortars at 28 days (P0R1.5, P0R2, and P0R2.5), the drying shrinkage of P20R1.5 decreased by 67.63%. Similarly, drying shrinkage of P20R2 increased by 54.25% and that of P20R2.5 decreased by 26.07%. This decrease is attributed to the geopolymer gel which became denser and resulted in porosity reduction. The curing of geopolymer mortar at the temperature of 60 °C helped in improving the geopolymerization reaction and results in a dense structure.

### 3.6. Microstructural Analysis

#### 3.6.1. Field Emission Scanning Microscopy (FESEM)

**Figure 8:**
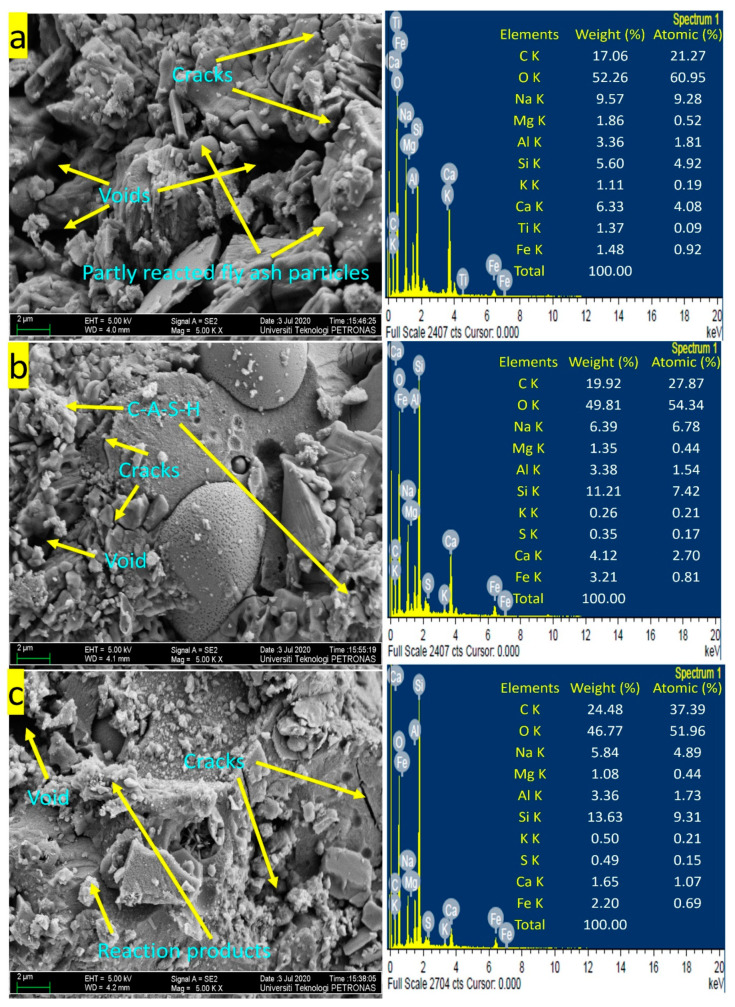
FESEM images with EDX (**a**) P0R2, (**b**) P10R2, (**c**) P20R2 geopolymer mortar.

FESEM investigation aims to study the dispersion of PSA in the geopolymer mortars. The morphology of samples of geopolymer mortar made with PSA addition and a reference mortar (without PSA) with their energy-dispersive X-ray (EDX) were examined using FESEM and illustrated in Figure 8. The content of PSA within the geopolymer mortar mixtures was 10%, and 20% replacement for fly ash by mass, with 2 as the ratio of NS to NH. FESEM investigation aims to study the dispersion of PSA in the geopolymer mortars. From the micrographs, it is evident that the aluminosilicate gel (glassy phase), voids, cracks, and several fully and partially reacted particles of fly ash that were embedded in the glassy phase can be seen. The appearance of cracks in the geopolymer mortar is due to the gel drying shrinkage during the process of polycondensation that grows the capillary tension in the gel matrices. Moreover, this drying shrinkage might be attributed to nonuniform internal pressure and evaporating water.

The micrograph of P0R2 geopolymer mortar presented in Figure 8a revealed cracks, voids, and a uniform microstructure. The fly ash contains considerable spherical particles and hollow spheres. Partially dissolved particles formed spread voids in the matrices of the geopolymer mortar which is like the findings reported by Huseien et al. [66]. These partly reacted particles of fly ash were recognized in cavities that can be associated with the spaces of the dissolved fly ash constituent parts which resulted in lower compressive strength of 34.97 MPa at 28 days. Figure 8b showed the images of P10R2 geopolymer mortar containing 10% of PSA. This image indicated a more compact structure, fewer voids, and cracks than P0R2 geopolymer mortar. Thus, the UCS strength of P10R2 geopolymer mortar increased by 15.10%. Figure 8c illustrates the micrograph of P20R2 geopolymer mortar containing 20% of PSA. The FESEM image appeared to be compact, dense, and discretely uniform phase compared to P0R2 by 11.18%. The existence of compact structure in the geopolymer mortar samples indicated the presence of reaction products (calcium or sodium aluminosilicate hydrate, C, N–A–S–H gel) which yielded higher UCS strength than P0R2 geopolymer mortar.

The EDX results show different developments in the P0R2, P10R2, and P20R2 mortar matrix. From Figure 8a, the P0R2 geopolymer mortar revealed lower Si content of 5.60%, whereas the P10R2 (Figure 8b) and P20R2 (Figure 8c) geopolymer mortars have Si content of 11.21% and 13.63%. The geopolymer mortars P0R2, P10R2, and P20R2 have ratios of Al to Si as 0.60, 0.30, and 0.25; ratios of Ca to Si as 1.13, 0.37, and 0.12; and ratios of Ca to Na as 0.66, 0.64, and 0.28 respectively. These ratios of elements are attributed to C–A–S–H gel formations. Also, C, Mg, O, K, Fe, and S were the residues in the mortar matrices. During the process of reaction, the elements’ residues were not fully melted which has a composition and morphology.

#### 3.6.2. Mercury Intrusion Porosimetry

**Figure 9:**
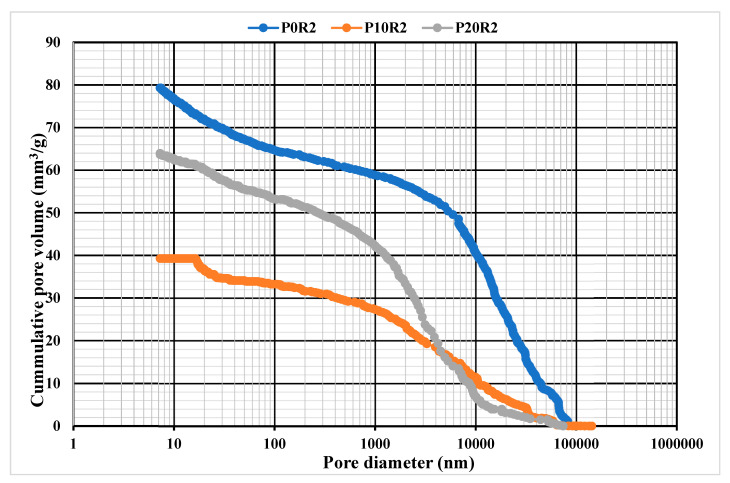
Pore size distribution for P0R2, P10R2, and P20R2 geopolymer mortar.

The porosity of the geopolymer mortar samples was determined with the aid of a mercury intrusion porosimetry (MIP) test. MIP test was conducted on the hardened P0R2, P10R2, and P20R2 geopolymer mortar samples and presented in Figure 9. The effect of PSA on the soundness of geopolymer mortar may be determined in terms of the total volume of the pores, decreased or increased distribution of pore diameter, and average accessible porosity. From Figure 9, the cumulative pore volume for P0R2, P10R2, and P20R2 are 78.59, 39.25, and 63.50 mm^3^/g respectively. Compared with P0R2, the inclusion of the 10 wt % of PSA (P10R2) and 20 wt % of PSA (P20R2), the total pore volume notably decreased by 50% and 19.20% respectively. This confirms the addition of PSA in the geopolymer matrices lowered the cumulative porosity when compared to the reference mortar (P0R2). This is attributed to the result of the filling device of the voids and pores by the PSA serving as a strengthening factor that densified the matrices of geopolymer. The cumulative pore volumes of the three geopolymer mixtures are in line with the result of compressive strengths and FESEM images of the mixtures. Also, remarkable index parameters defining the characteristics of the penetrable structure are stated in Table 5. The average pore diameter, the total surface area of the pores, and the median pore diameter measured by the volume change of the mercury penetration, and the modal pore diameter determined by the difference of volume of the pores are concerning the variation in strength. From Table 5, the average pore diameter is found to be between 118.66 and 135.90 nm. The improvement of the geopolymerization reaction at the age of 28 days enhanced the pores which contribute to a thicker structure. At a later period, the improvement of the geopolymerization reaction refined the pores and led to a denser structure [67].

### 3.7. Polycyclic Aromatic Hydrocarbons in Petroleum Sludge Ash and Geopolymer Mortar

Polycyclic aromatic hydrocarbons (PAHs) consist of a large group of stable compounds of hydrophobic organic which are very persistent in the environment. Sixteen of the PAHs were enumerated as priority pollutants by the United States Environmental Protection Agency (USEPA) because of their toxic, mutagenic, and carcinogenic effects. PAHs of PSA and crushed geopolymer mortar particles P10R2 and P20R2 mixture were determined. From Table 6, the 16 PAHs analyzed were found to be within the permissible limit of the USEPA.

### 3.8. Statistical Interpretation of the Test Results

All the developed models were statistically analyzed as well as validated accordingly. The analyses were conducted at a 5% significance level to study the importance of variables of the experiment. Flexural strength and water absorption of the geopolymer mortars were considered as the output variables in the present study, whereas the amount of PSA and ratio of Na_2_SiO_3_ to NaOH (NS: NH) were carefully chosen as independent variables.

The terms of the models are significant terms possessing *p*-values of <0.05 which indicates that the terms are relevant for the models of output variables at a confidence level of 95% and recognized as key parameters on the test results, whereas the other terms were not much relevant as their *p*-values were >0.05 and this implied that the terms have less influence upon the flexural and water absorption of the geopolymer mortar. The fitness and quality of the established model might be described by its high level of correlation. The quality of the models could be measured using a lack of fit; the lesser the lack of fit value shows models of worthiness. As shown in Table 7, the lack of fit *p*-values for all the models is greater than 0.05 signified that the lack of fit was insignificant and consequently indicated that the response models were excellently fit. The models’ quality could also be examined through the R^2^ value. As shown in Table 8, the high R^2^ values of 0.9223 and 0.9647 for flexural strength and water absorption model indicating a good measure of correspondence between the predicted and experimental results. It is also worth mentioning that the predicted R^2^ values are in good agreement with the adjusted R^2^ values because the difference between them is less than 0.2. As illustrated in Table 8, all models have enough precision values of more than 4, signifying that the models could be used to navigate the design space. The final models’ equations for the output variables of the geopolymer mortar with all the model terms are presented in Equations (5) and (6). Using the analysis of variance model equations, the flexural strength and water absorption of the geopolymer mortar can be estimated.

Flexural strength = + 1.16872 + 0.074333A + 1.68667B(5)

Water absorption = + 6.44628 + 8.3333 × 10^−4^A − 0.8700B (6)

**Figure 10:**
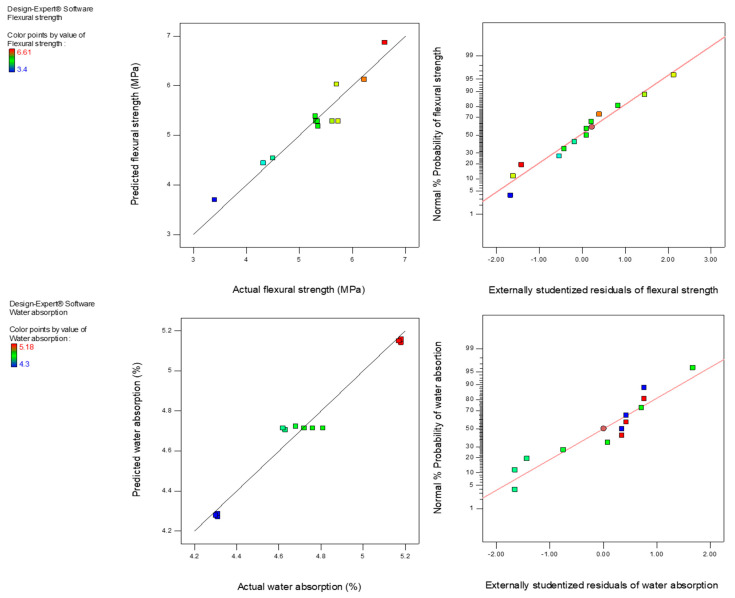
Predicted vs. actual and normal plot of residuals for output variables.

Plots of predicted vs. actual results of response variables graphically studied the competency as well as fitness of the flexural strength and water absorption. In Figure 10, the predicted vs. actual results plot revealed that the predicted response models were precise. The points are fitted smoothly along a straight line showing a good relationship in the established models among experimental and predicted results. Thus, the established models of the response variables were relevant and appropriate in estimating the flexural strength and water absorption of the geopolymer mortar. The normal probability plot is a graphical representation used to assess the data distribution and confirm its sufficiency. The points were distributed almost along the straight line of equality for all the dependent variables and, thus, showed that the data were normally distributed for all residual responses.

### 3.9. Optimization and Validation Study

**Figure 11:**
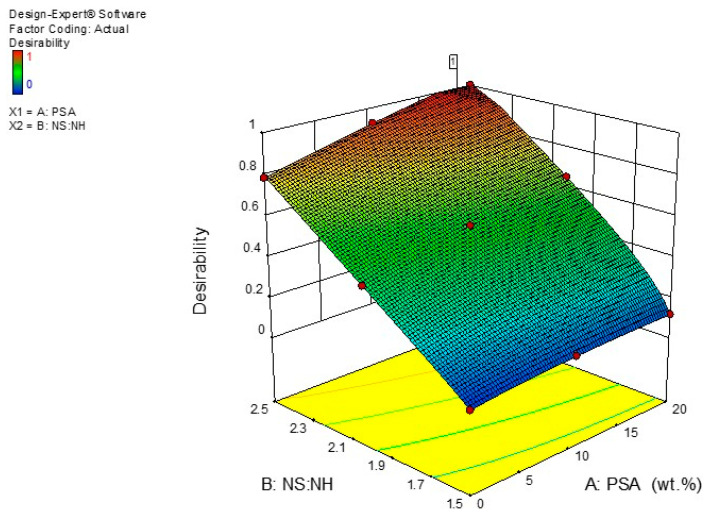
Desirable combination of PSA with the ratio of sodium silicate to sodium hydroxide.

**Figure 12:**
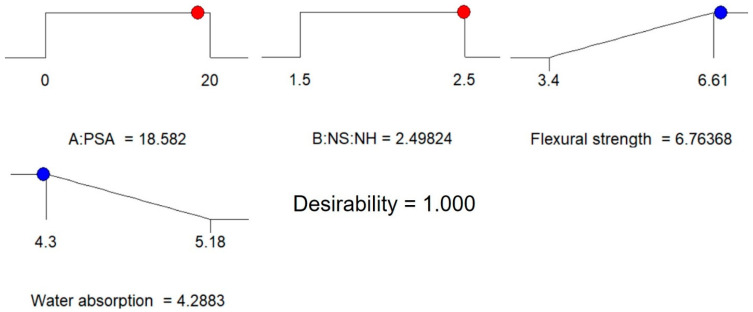
Optimization ramps of the geopolymer mortar.

Numerical multi-objective optimization was employed to define the optimum amount of PSA and the ratio of sodium silicate to sodium hydroxide to maximize the flexural strength and minimize the water absorption of the developed geopolymer mortar. The optimization study focused on recognizing the accepted values of input variables to accomplish the optimization goals. The responses influenced by the multiple factors were improved using the RSM method, which explained the significant function for the targeted output variables to enhance the responses. According to the optimization goals, the principles for optimization are summed in Table 9. The design expert software obtained the maximum desired mixture fractions through synthesizing 18.582 wt % of PSA with 2.5 as the ratio of sodium silicate to sodium hydroxide. The improved responses with associated desirability of 100% were being achieved. The numerical optimization results for the developed models were shown in the 3-D plot depicted in Figure 11. Besides, the independent factors and outputs were displayed graphically by the optimization ramps depicted in Figure 12.

To verify the fitness of the optimization effects and the whole response models, a supplementary set of examinations was conducted applying the optimized mixture relationships and two more separate mixes to confirm the optimized mix proportion. The error in the laboratory and predicted values were assessed using Equation (7). The percentage error and the predicted results, as well as experimental results, were presented in Table 10. According to the calculated percentage errors, it can be concluded that the experimental and predicted results were in excellent agreement with one another due to the percentage errors are reasonably small.

(7) $$Error\left(\%\right)=\frac{Experimental\;model-predicted\;model}{Experimental\;model}\times 100\%$$

## 4. Conclusions

Fly ash-based geopolymer mortars with PSA addition were designed and produced with the help of the RSM statistical tool. Mechanical and microstructural properties of the geopolymer mortar were investigated. Based on the findings, the following conclusions were made:

- At 28 days, the density of P0R2 was found to be higher than the densities of P10R2 and P20R2 by 3.21% and 18.61% respectively. The 28 days compressive strengths of P10R2 and P20R2 were found to be higher than that of the control geopolymer mortar (P0R2) by 13.11% and 10.05% respectively.
- Flexural strength increased linearly as the PSA content and ratio of NS to NH increased. The water absorption decreased linearly with an increase in the ratio of sodium silicate to sodium hydroxide (NS: NH) and PSA content.
- The mix combination of 10 wt % and 2.5 as the ratio of sodium silicate to sodium hydroxide (P10R2.5) yielded the best drying shrinkage result.
- The micrograph and pore size distribution analysis showed incorporating 10 wt % of PSA in the geopolymer mortar caused structural aluminosilicate modifications. The modifications were associated with the geopolymerization reaction of reactive silica and alumina in the precursor materials and alkaline solution. The polycyclic aromatic hydrocarbons were found to be within the permissible limit of USEPA.
- Experimental results were close to the validation results obtained and the numerical optimizations indicated that the optimum results could be achieved using 2.5 as the ratio of sodium silicate to sodium hydroxide, replacing 18.582 wt % of fly ash with PSA. PSA and FA as precursor materials can reduce carbon dioxide emission, disposal of waste materials. The geopolymer mortar could be used as a repair material in the building industries.

## Figures and Tables

**Table 1:** Chemical composition of PSA and fly ash (%).

	Al_2_O_3_	SiO_2_	Fe_2_O_3_	CaO	MgO	P_2_O_5_	SO_3_	K_2_O	TiO_2_	Others	LOI	Blaine Fineness (m^2^/kg)	Specific Gravity
PSA	10.00	14.90	45.90	9.26	2.41	1.75	11.5	1.08	0.41	2.70	0.09	117	2.35
FA	17.40	36.40	20.20	14.5	2.40	1.27	2.01	2.31	1.59	1.64	0.28	384	2.68

**Table 2:** RSM boundaries of variables.

Factors	Code	Units	Levels		
			−1	0	+1
PSA	A	%	0	10	20
NS: NH	B		1.5	2.0	2.5

**Table 3:** Mix formulations of modified geopolymer mortar.

Mixtures	P20R2	P10R2	P10R2	P20R1.5	P0R2	P0R1.5	P10R2	P20R2.5	P10R2	P10R2	P10R1.5	P10R2.5	P0R2.5
A: PSA (%)	20	10	10	20	0	0	10	20	10	10	10	10	0
B: NS: NH	2	2	2	1.5	2	1.5	2	2.5	2	2	1.5	2.5	2.5

**Table 4:** Mixture proportion of geopolymer mortar.

Sand (kg/m^3^)	Alkaline Solution (kg/m^3^)	PSA (%)	Fly Ash (kg/m^3^)	PSA Content (kg/m^3^)
1314.29	328.57	0	657.14	0
1314.29	328.57	10	591.43	65.71
1314.29	328.57	20	525.71	131.43

**Table 5:** Pore structure properties of geopolymer mortar.

Mixture	Modal Pore Diameter D10 (nm)	Median Pore Diameter D90 (nm)	Average Pore Diameter D50 (nm)	Total Surface Area (m^2^/g)	Porosity (%)
P0R2	11,746.79	13,619.79	118.66	2.355	12.89
P10R2	16.69	3166.26	135.90	1.155	0.25
P20R2	18,151.37	2418.80	119.93	2.017	13.01

**Table 6:** Polycyclic aromatics hydrocarbons (PAHs) mg/kg.

Parameters	Abbreviation	No. of PAHs Rings	PSA	P10R2	P20R2	Permissible Limits [68]
Naphthalene	Nap	2	<0.5	<0.5	<0.5	5.52
Acenaphthylene	Acy	3	<0.5	<0.5	<0.5	NA
Acenaphthene	Ace	3	<0.5	<0.5	<0.5	3590
Fluorene	Fle	3	<0.5	<0.5	<0.5	2390
Phenanthrene	Phe	3	1.9	<0.5	<0.5	NA
Anthracene	Ant	3	<0.5	<0.5	<0.5	17,900
Fluoranthene	Fla	4	<0.5	<0.5	<0.5	2390
Pyrene	Pyr	4	<0.5	<0.5	<0.5	1790
Benz(a)anthracene	BaA	4	<0.5	<0.5	<0.5	1.14
Chrysene	Chr	4	<0.5	<0.5	<0.5	115
Benzo(b)fluoranthene	BbF	5	<1.0	<1.0	<1.0	1.15
Benzo(k)fluoranthene	BkF	5	<1.0	<1.0	<1.0	11.5
Benzo(a)pyrene	BaP	5	<0.05	<0.05	<0.05	0.115
Indeno(1.2.3.cd)pyrene	IcdP	6	<0.5	<0.5	<0.5	1.15
Dibenzo(a.h)anthracene	DahA	5	<0.5	<0.5	<0.5	0.115
Benzo(g.h.i)perylene	BghiP	6	<0.5	<0.5	<0.5	NA

**Table 7:** Analysis of variance for response models.

Responses	Factors	SS	Df	MS	F-Value	*p*-Value	Remarks
Flexural strength (MPa)	Model	7.58	2	3.79	59.31	<0.0001	Significant
	A-PSA	3.32	1	3.32	51.87	<0.0001	
	B- NS: NH	4.27	1	4.27	66.76	<0.0001	
	Lack of Fit	0.48	6	0.080	2.03	0.2567	Not significant
Water absorption (%)	Model	1.14	2	0.57	136.65	<0.0001	Significant
	A-PSA	4.167 × 10^−4^	1	4.167 × 10^−4^	0.10	0.7580	
	B- NS: NH	1.14	1	1.14	273.21	<0.0001	
	Lack of Fit	0.013	6	2.139 × 10^−3^	0.30	0.9094	Not significant

Where SS: the sum of squares; Df: the degree of freedom, P: Probability; F: Fisher statistical value; MS: mean square.

**Table 8:** Validation properties of response models.

Response variable	SD	Mean	R^2^	Adj. R^2^	Pred. R^2^	AP
Flexural strength (MPa)	0.25	5.29	0.9223	0.9067	0.8597	26.128
Water absorption (%)	0.064	4.71	0.9647	0.9576	0.9487	28.632

Where SD: Standard deviation, R^2^: correlation coefficient, Adj. R^2^: adjusted correlation coefficient, Pred. R^2^: predicted R^2^, AP: adequate precision.

**Table 9:** Optimization benchmarks.

Variables and Responses	Unit	Goals	Lower Limit	Upper Limit
PSA	%	In range	0	20
NS to NH ratio		In range	1.5	2.5
Flexural strength (MPa)	MPa	Maximize	3.4	6.61
Water absorption	(%)	Minimize	4.3	5.18

**Table 10:** Model validation.

Responses	PSA (%)	NS To NH Ratio	Predicted Outcomes	Experimental Outcomes	Error (%)
Flexural strength (MPa)	18.58	2.5	6.76	6.90	2.03
	10	2	5.29	5.61	5.70
	20	2	6.03	5.70	5.79
Water absorption (%)	18.58	2.5	4.29	4.48	4.24
	10	2	4.71	5.0	5.8
	20	2	4.72	5.0	5.6

## Data Availability

Not applicable.

## References

[1] Mohammed B.S., Haruna S., Mubarak bn Abdul Wahab M., Liew M.S. (2019). Optimization and characterization of cast in-situ alkali-activated pastes by response surface methodology. Constr. Build. Mater..

[2] Rakhimova N.R., Rakhimov R.Z. (2019). Literature Review of Advances in Materials Used in Development of Alkali-Activated Mortars, Concretes, and Composites. J. Mater. Civ. Eng..

[3] Amran Y.H.M., Alyousef R., Alabduljabbar H., El-Zeadani M. (2020). Clean production and properties of geopolymer concrete; A review. J. Clean. Prod..

[4] Ranjbar N., Zhang M. (2020). Fiber-reinforced geopolymer composites: A review. Cem. Concr. Compos..

[5] Scrivener K.L., John V.M., Gartner E.M. (2018). Eco-efficient cements: Potential economically viable solutions for a low-CO_2_ cement-based materials industry. Cem. Concr. Res..

[6] Bhutta A., Farooq M., Zanotti C., Banthia N. (2016). Pull-out behavior of different fibers in geopolymer mortars: Effects of alkaline solution concentration and curing. Mater. Struct..

[7] Castel A., Foster S.J. (2015). Bond strength between blended slag and Class F fly ash geopolymer concrete with steel reinforcement. Cem. Concr. Res..

[8] Dhakal S. (2009). Urban energy use and carbon emissions from cities in China and policy implications. Energy Policy.

[9] Shalini A., Gurunarayanan G., Arun kumar R., Jaya prakash V., Sakthivel S. (2016). Performance of Rice Husk Ash in Geopolymer Concrete. Int. J. Innov. Res. Sci. Technol..

[10] Bhikshma V., Koti R.M., Srinivas R.T. (2012). An Experimental Investigation on Properties of Geopolymer Concrete (No Cement Concrete). Asian J. Civ. Eng. (Build. Hous.).

[11] Nurruddin M.F., Haruna S., Mohammed B.S., Shaaban I.G. (2018). Methods of curing geopolymer concrete: A review. Int. J. Adv. Appl. Sci..

[12] Mohammed B.S., Haruna S., Wahab M.M.A., Liew M.S., Haruna A. (2019). Mechanical and microstructural properties of high calcium fly ash one-part geopolymer cement made with granular activator. Heliyon.

[13] Duxson P., Fernández-Jiménez A., Provis J.L., Lukey G.C., Palomo A., van Deventer J.S.J. (2006). Geopolymer technology: The current state of the art. J. Mater. Sci..

[14] Colangelo F., Farina I., Travaglioni M., Salzano C., Cioffi R., Petrillo A. (2021). Eco-efficient industrial waste recycling for the manufacturing of fibre reinforced innovative geopolymer mortars: Integrated waste management and green product development through LCA. J. Clean. Prod..

[15] Zhang Y., Xiao R., Jiang X., Li W., Zhu X., Huang B. (2020). Effect of particle size and curing temperature on mechanical and microstructural properties of waste glass-slag-based and waste glass-fly ash-based geopolymers. J. Clean. Prod..

[16] Roviello G., Ricciotti L., Tarallo O., Ferone C., Colangelo F., Roviello V., Cioffi R. (2016). Innovative Fly Ash Geopolymer-Epoxy Composites: Preparation, Microstructure and Mechanical Properties. Materials.

[17] Mehta A., Siddique R. (2016). An overview of geopolymers derived from industrial by-products. Constr. Build. Mater..

[18] Zhou W., Shi X., Lu X., Qi C., Luan B., Liu F. (2020). The mechanical and microstructural properties of refuse mudstone-GGBS-red mud based geopolymer composites made with sand. Constr. Build. Mater..

[19] Bonet-Martínez E., Pérez-Villarejo L., Eliche-Quesada D., Carrasco-Hurtado B., Bueno-Rodríguez S., Castro-Galiano E. (2018). Inorganic polymers synthesized using biomass ashes-red mud as precursors based on clay-kaolinite system. Mater. Lett..

[20] Rakhimova N.R., Rakhimov Ravil Z., Morozov Vladimir P., Gaifullin Albert R., Potapova Ludmila I., Gubaidullina Alfiya M., Osin Yury N. (2018). Marl-based geopolymers incorporated with limestone: A feasibility study. J. Non-Cryst. Solids.

[21] Kurda R., Silva R.V., de Brito J. (2020). Incorporation of Alkali-Activated Municipal Solid Waste Incinerator Bottom Ash in Mortar and Concrete: A Critical Review. Materials.

[22] Abubakr A.E., Soliman A.M., Diab S.H. (2020). Effect of activator nature on the impact behaviour of Alkali-Activated slag mortar. Constr. Build. Mater..

[23] Ramirez D., Collins C.D. (2018). Maximisation of oil recovery from an oil-water separator sludge: Influence of type, concentration, and application ratio of surfactants. Waste Manag..

[24] Fitri I., Ni’matuzahroh, Surtiningsih T. (2017). Bioremediation of oil sludge using a type of nitrogen source and the consortium of bacteria with composting method. AIP Conf. Proc. AIP Publ. LLC.

[25] Nazem M.A., Tavakoli O. (2017). Bio-oil production from refinery oily sludge using hydrothermal liquefaction technology. J. Supercrit. Fluids.

[26] Xu M.X., Wang H.X., Ouyang H.D., Zhao L., Lu Q. (2021). Direct catalytic decomposition of N_2_O over bismuth modified NiO catalysts. J. Hazard. Mater..

[27] Chen G., Cheng C., Zhang J., Sun Y., Hu Q., Qu C., Dong S. (2019). Synergistic effect of surfactant and alkali on the treatment of oil sludge. J. Pet. Sci. Eng..

[28] Mubarak Kankia U., Lavania B., Bashar S.M., Suhaimi B.H., Effa Affiana I., Zakariyya Zango U. (2020). Review of petroleum sludge thermal treatment and utilization of ash as a construction material, a way to environmental sustainability. Int. J. Adv. Appl. Sci..

[29] Wang X., Wang Q., Wang S., Li F., Guo G. (2012). Effect of biostimulation on community level physiological profiles of microorganisms in field-scale biopiles composed of aged oil sludge. Bioresour. Technol..

[30] Aguelmous A., El Fels L., Souabi S., Zamama M., Yasri A., Lebrihi A., Hafidi M. (2018). Petroleum sludge bioremediation and its toxicity removal by landfill in gunder semi-arid conditions. Ecotoxicol. Environ. Saf..

[31] Lin B., Huang Q., Chi Y. (2018). Co-pyrolysis of oily sludge and rice husk for improving pyrolysis oil quality. Fuel Process. Technol..

[32] Lin B., Wang J., Huang Q., Chi Y. (2017). Effects of potassium hydroxide on the catalytic pyrolysis of oily sludge for high-quality oil product. Fuel.

[33] Shen Y., Chen X., Wang J., Ge X., Chen M. (2016). Oil sludge recycling by ash-catalyzed pyrolysis-reforming processes. Fuel.

[34] da Silva L.J., Alves F.C., de Franca F.P. (2012). A review of the technological solutions for the treatment of oily sludges from petroleum refineries. Waste Manag. Res..

[35] Johnson O.A., Affam A.C. (2019). Petroleum sludge treatment and disposal: A review. Environ. Eng. Res..

[36] Gong Z., Liu L., Zhang H., Wang Z., Wu J., Guo Y., Zhang J. (2020). Study on the Migration Characteristics of As, Pb, and Ni during Oily Sludge Incineration with CaO Additive. Energy Fuels.

[37] Pinheiro B.C.A., Holanda J.N.F. (2009). Processing of red ceramics incorporated with encapsulated petroleum waste. J. Mater. Process. Technol..

[38] Pinheiro B.C., Holanda J.N. (2013). Reuse of solid petroleum waste in the manufacture of porcelain stoneware tile. J. Environ. Manag..

[39] Xiao W., Yao X., Zhang F. (2019). Recycling of Oily Sludge as a Roadbed Material Utilizing Phosphogypsum-Based Cementitious Materials. Adv. Civ. Eng..

[40] Souza A.J., Pinheiro B.C.A., Holanda J.N.F. (2011). Valorization of Solid Petroleum Waste as a Potential Raw Material for Clay-Based Ceramics. Waste Biomass Valorization.

[41] Sengupta P., Saikia N., Borthakur P.C. (2002). Bricks from Petroleum Effluent Treatment Plant Sludge: Properties and Environmental Characteristics. J. Environ. Eng..

[42] Pakpahan E.N., Shafiq N., Isa M.H., Kutty S.R.M., Mustafa M.R. (2016). Petroleum sludge thermal treatment and use in cement replacement—A solution towards sustainability. Engineering Challenges for Sustainable Future—Proceedings of the 3rd International Conference on Civil, Offshore and Environmental Engineering, ICCOEE 2016, Kuala Lumpur, Malaysia, 15–17 August 2016.

[43] Usman K.M., Baloo L., Mohammed B.S., Hassan S.B., Haruna S., Danlami N., Affiana Ishak E., Nurliyana Samahani W. (2021). Effects of petroleum sludge ash in fly ash-based geopolymer mortar. Constr. Build. Mater..

[44] Hernandez A.B., Ferrasse J.H., Chaurand P., Saveyn H., Borschneck D., Roche N. (2011). Mineralogy and leachability of gasified sewage sludge solid residues. J. Hazard. Mater..

[45] ASTM Committee (2010). Standard Specification for Coal Fly Ash and Raw or Calcined Natural Pozzolan for Use.

[46] ASTM (2013). Standard Specification for Concrete Aggregates.

[47] Bakharev T., Sanjayan J.G., Cheng Y.-B. (1999). Alkali activation of Australian slag cements. Cem. Concr. Res..

[48] Karakoç M.B., Türkmen İ., Maraş M.M., Kantarci F., Demirboğa R., Uğur Toprak M. (2014). Mechanical properties and setting time of ferrochrome slag based geopolymer paste and mortar. Constr. Build. Mater..

[49] ASTM (2013). ASTM C109/109M. Standard Test Method for compressive Strength of Hydraulic Cement Mortars (Using 2-In. or [50-mm] Cube Specimens).

[50] ASTM (2016). ASTM C293/C293M-16. Standard Test Method for Flexural Strength of Concrete (Using Simple Beam with Center-Point Loading).

[51] ASTM (2017). ASTM C157/C157M-17. Standard Test Method for Length Change of Hardened Hydraulic-Cement Mortar and Concrete.

[52] ASTM (2013). ASTM C642-13. Standard Test Method for Density, Absorption, and Voids in Hardened Concrete.

[53] USEPA (1996). Method 3540C. Soxhlet extraction. SW-846.

[54] Yahya Z., Abdullah M.M.A.B., Talib S.Z.A., Razak R.A. (2017). Comparative study on early strength of sodium hydroxide (NaOH) activated fly ash based geopolymer. AIP Conf. Proc. AIP Publ. LLC.

[55] Hwang C.-L., Huynh T.-P. (2015). Effect of alkali-activator and rice husk ash content on strength development of fly ash and residual rice husk ash-based geopolymers. Constr. Build. Mater..

[56] Songpiriyakij S., Kubprasit T., Jaturapitakkul C., Chindaprasirt P. (2010). Compressive strength and degree of reaction of biomass- and fly ash-based geopolymer. Constr. Build. Mater..

[57] Ranjbar N., Mehrali M., Alengaram U.J., Metselaar H.S.C., Jumaat M.Z. (2014). Compressive strength and microstructural analysis of fly ash/palm oil fuel ash based geopolymer mortar under elevated temperatures. Constr. Build. Mater..

[58] Kupaei R.H., Alengaram U.J., Jumaat M.Z.B., Nikraz H. (2013). Mix design for fly ash based oil palm shell geopolymer lightweight concrete. Constr. Build. Mater..

[59] Atabey İ.İ., Karahan O., Bilim C., Atiş C.D. (2020). The influence of activator type and quantity on the transport properties of class F fly ash geopolymer. Constr. Build. Mater..

[60] Noushini A., Castel A. (2016). The effect of heat-curing on transport properties of low-calcium fly ash-based geopolymer concrete. Constr. Build. Mater..

[61] Zhang Z., Provis J.L., Reid A., Wang H. (2014). Geopolymer foam concrete: An emerging material for sustainable construction. Constr. Build. Mater..

[62] Abdollahnejad Z., Mastali M., Mastali M., Dalvand A. (2017). Comparative Study on the Effects of Recycled Glass–Fiber on Drying Shrinkage Rate and Mechanical Properties of the Self-Compacting Mortar and Fly Ash–Slag Geopolymer Mortar. J. Mater. Civ. Eng..

[63] Si R., Dai Q., Guo S., Wang J. (2020). Mechanical property, nanopore structure and drying shrinkage of metakaolin-based geopolymer with waste glass powder. J. Clean. Prod..

[64] Yan S., Sagoe-Crentsil K. (2012). Properties of wastepaper sludge in geopolymer mortars for masonry applications. J. Env. Manag..

[65] Priyadharshini P., Ramamurthy K., Robinson R.G. (2017). Excavated soil waste as fine aggregate in fly ash based geopolymer mortar. Appl. Clay Sci..

[66] Huseien G.F., Sam A.R.M., Mirza J., Tahir M.M., Asaad M.A., Ismail M., Shah K.W. (2018). Waste ceramic powder incorporated alkali activated mortars exposed to elevated Temperatures: Performance evaluation. Constr. Build. Mater..

[67] Haruna S., Mohammed Bashar S., Wahab M.M.A., Liew M.S. (2020). Effect of paste aggregate ratio and curing methods on the performance of one-part alkali-activated concrete. Constr. Build. Mater..

[68] Siemering G.S., Thiboldeaux R. (2021). Background concentration, risk assessment and regulatory threshold development: Polycyclic aromatic hydrocarbons (PAH) in Milwaukee, Wisconsin surface soils. Environ. Pollut..

